# Factors Related to Hypertension in Pediatric Patients Who Do Not Have Obstructive Sleep Apnea: A Retrospective Chart Study

**DOI:** 10.3390/jcm14134699

**Published:** 2025-07-03

**Authors:** Alyssa Exarchakis, Alexandra Cohen, Penghao Wang, Seema Rani, Diana Martinez

**Affiliations:** 1 Department of Biomedical Sciences, Cooper Medical School, Rowan University, 401 South Broadway, Camden, NJ 08103, USA; exarch47@rowan.edu (A.E.); wangpeng@rowan.edu (P.W.); 2 Sidney Kimmel Medical College, Thomas Jefferson University, Philadelphia, PA 19107, USA; alexandra.cohen@students.jefferson.edu; 3 Nemours Children’s Hospital, 1600 Rockland Road, Wilmington, DE 19803, USA; seema.rani@nemours.org

**Keywords:** polysomnography, mental illness, neurological disease, non-OSA, respiratory disease

## Abstract

**Background/Objectives**: The relationship between OSA and adult hypertension has been extensively studied; however, it remains understudied in pediatric patients without OSA. The aim of this study is to identify factors associated with pediatric hypertension without OSA, through an IRB-approved retrospective chart review of patients who underwent polysomnography at Nemours Children’s Hospital, DE/NJ between January 2020 and July 2023. **Methods**: Eligibility criteria included children 8–17 years, completed PSG, and clinic visit blood pressure (BP). Anthropometrics, demographics, social determinants, and medical history were obtained from electronic medical records. Hypertension was defined as the average systolic and/or diastolic BP that is ≥95th percentile for gender, age, and height based on AAP Clinical Practice Guidelines. All variables were checked for normality. Chi-square tests for categorical data and Wilcoxon rank sum tests for continuous data were used to test significance between non-OSA non-hypertensives (NH) and hypertensives (H). *p* < 0.05 is considered significant. **Results:** Of 285 charts evaluated, 137 were classified as non-OSA. Patient information, including parents in household, smoking exposure, and food allergies, were statistically significant (*p* < 0.05) in hypertensive pediatric patients without OSA. Hypertension was significantly correlated (*p* < 0.05) with birth weight, BMI, daytime heart rate, systolic BP, and diastolic BP. Statistically significant differences (*p* < 0.05) were found in mental illnesses, neurological disease, and respiratory disease. Among polysomnography parameters, only nighttime heart rate was found to be statistically significant. **Conclusions:** The data suggests that in pediatric patients without OSA, there are multiple factors and co-morbidities associated with hypertension. These factors and co-morbidities warrant additional follow up in clinical practice to mitigate the risks of hypertension in pediatric patients.

## 1. Introduction

Hypertension is the leading preventable risk factor for cardiovascular disease (CVD) and all-cause mortality worldwide in adults [1,2]. The risk factors for hypertension and its consequence on adult cardiovascular health are heavily studied areas. However, unlike the adult population, the risk factors associated with hypertension in the pediatric population are understudied.

Hypertension is not an uncommon disease in children, with a reported global prevalence of 4% [3]. Height, sex, and age are important determinants of pediatric blood pressure (BP). Increasing evidence suggests that high blood pressure from childhood persists to adulthood, suggesting children with high blood pressure are more likely to develop hypertension later in life [4]. Traditional risk factors for pediatric hypertension include male sex, older age, obesity, high sodium intake, ethnicity, family history of hypertension, poor sleep quality, and more [5,6]. Some risk factors are modifiable and some are not modifiable. Modifiable risk factors include: diet, sleep disorders (sleep disordered breathing and OSA), BMI, medications, and physical activity. Non-modifiable risk factors include: socioeconomic status, genetics, family history (parental hypertension, birth weight, smoke exposure), gender, endocrine issues, racial/ethnicity predisposition, and mental illness [7,8,9,10,11,12]. A cross-sectional observational study that evaluated children who were recruited sequentially from the Pediatric Sleep Disorders Clinic at Johns Hopkins Hospital for evaluation of snoring and difficulty breathing during sleep found that children with Obstructive Sleep Apnea (OSA) had a significantly higher diastolic BP than those with primary snoring [13]. OSA is a sleep disorder that can occur at all ages and is associated with intermittent, near complete or partial reduction of airflow during sleep. In adults with OSA, symptoms typically include daytime somnolence, whereas children with OSA are more likely to present with behavioral and cognitive disorders, including hyperactivity, attention-deficit disorder, poor school performance, and nocturnal enuresis [14]. The gold standard test for diagnosing OSA in children is an overnight attended in-laboratory polysomnogram (PSG). According to the 2012 American Academy of Sleep Medicine scoring manual, the criteria for events during sleep for infants and children can be used for those 18 years and younger, but these can vary, and sleep laboratory scored using adult criteria in children.

The relationship between OSA and hypertension has been extensively studied; however, very few studies have investigated hypertension in non-OSA pediatric patients. Prior studies have shown that sympathetic activation from sleep disorders may have implications on cardiovascular health [15,16], including elevated blood pressure. Javaheri et al. carried out a cross-sectional study to examine the relationship between insufficient sleep and pre-hypertension in healthy adolescents. After adjusting for the same variables, they found that adolescents with a sleep efficiency of ≤85%, as measured by polysomnography, had nearly three times the odds of having pre-hypertension compared to those with higher sleep efficiency. Studies show that people from low-income families or racial and ethnic minority groups are more likely to have poor sleep, which adds to health differences between groups [17,18,19]. People with lower income often have less regular sleep schedules [20]. Problems at home—like too many people in one space, loud noise, or feeling unsafe—can also make it harder to sleep well [21]. These conditions can keep families from getting enough rest or sticking to a steady sleep routine.

While the association between OSA and pediatric hypertension is well-documented, far less is known about the impact of non-apneic sleep disturbances on blood pressure in children. In this retrospective chart review, the aim is to investigate the understudied relationship between sleep parameters derived from polysomnography (PSG), medical history, and socioeconomic factors in pediatric patients with hypertension who do not have obstructive sleep apnea (OSA). The primary objective is to identify specific sleep characteristics, socioeconomic indicators, and comorbid medical conditions that are associated with elevated blood pressure in children without OSA. We hypothesize that poor sleep quality—independent of OSA—contributes to the development of hypertension in the pediatric population. This study addresses a critical gap by exploring whether the absence of OSA may still play a significant role in pediatric hypertension. Identifying these associations may broaden our understanding of sleep-related contributors to cardiovascular risk in children and could inform more comprehensive screening and intervention strategies in clinical practice. In the absence of OSA, hypertension was significantly correlated with birth weight, BMI z-score, daytime heart rate, systolic BP, and diastolic BP. Statistically significant differences were found in mental illnesses, neurological disease, and respiratory disease. This suggests that the development of hypertension in pediatric patients can occur due to several factors outside of OSA.

## 2. Materials and Methods

**Figure 1:**
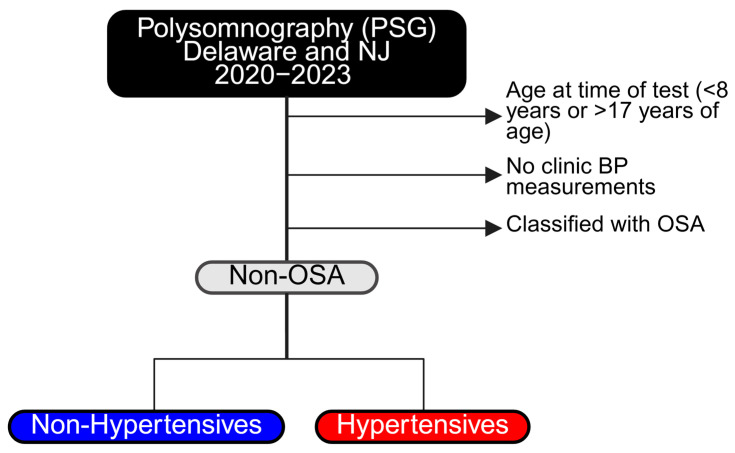
Flowchart for sample section: inclusion and exclusion criteria.

This is an IRB-approved retrospective chart review, completed July 2023–April 2024 from pediatric patients at Nemours Children’s Hospital who underwent polysomnography (PSG) in either the Wilmington, DE or Deptford, NJ locations between January 2020 and May 2023. This study was deemed exempt from informed consent by the Nemours Children’s Hospital Institutional Review Board and did not require participant consent. Eligibility criteria included children ages 8–17 years, regardless of gender, who had completed PSG and had office visit blood pressure recordings (Figure 1). Electronic medical records were reviewed for demographics and clinical data. Charts from 285 patients aged at least 8 years old and no older than 17 at the time of PSG were analyzed. Inter-rater reliability was ensured by frequent meetings that included developing clear operational definitions for information gathering. In the event of ambiguity, a meeting was conferred prior to continuing to allow for discussion and clarification. Charts were separated into OSA and non-OSA groups. Those without OSA were selected for future evaluation. OSA classification was based on PSG results according to the 2012 AASM scoring manual. Pediatric scoring rules for apnea and hypopnea and the categorization for OSA severity can be found here [14].

### 2.1. Polysomnography Testing

Data was collected from the overnight PSG in an American Academy of Sleep Medicine-accredited sleep laboratory at either the Nemours Children’s Hospital in Wilmington, DE or Deptford, NJ. Electroencephalography, electrooculography, electromyography, and electrocardiography were continuously recorded throughout the PSG. Respiratory effort was measured via respiratory inductance plethysmography and oxygen saturation was measured via a finger probe on a pulse oximeter. A nasal pressure cannula and a thermistor measured airflow, and snoring was measured via a microphone. The patients were monitored by a polysomnographic technician during the entire duration of the study in a dark and comfortable environment. The raw data was reviewed and interpreted by a pediatric board-certified sleep medicine physician. Respiratory events were scored according to the American Academy of Sleep Medicine (AASM) guidelines using pediatric scoring [22]. The PSG data collected included respiratory and non-respiratory sleep parameters. Sleep architecture and non-respiratory parameters included the patient’s total sleep time (TST), sleep efficiency (SE), arousal index (AI), percent time spent in each sleep stage, rapid eye movement (REM) and non-rapid eye movement (non-REM stages N1, N2, N3), periodic limb movement index (PLMI, total number of periodic limb movements/h), wakefulness after sleep onset (WASO, total number of minutes awake after sleep onset) and average HR during sleep [23]. The respiratory parameters consisted of the obstructive apnea index (OAI), hypopnea index, apnea-hypopnea index (AHI), REM AHI, and gas exchange measures, baseline end-tidal carbon dioxide (ETCO_2_) levels, % TST ETCO_2_ > 50, hypoventilation, % of the TST snoring. Nighttime heart rate (HR) was also measured and recorded. For analysis, PSG sleep parameters were extracted from electronic medical records (EMR). The number of PSGs that patients had undergone and classified them as the initial or subsequent PSG testing were recorded.

OSA can be reported as mild, moderate, or severe. Mild OSA has an AHI score of 1.5–4.9, with an SpO_2_ < 90% for 2–5% of TST and >92 oxygen saturation nadir (%). Moderate OSA has an AHI score of 5–9; SpO_2_ < 90% for 5–10% of TST and >80 but <92 oxygen saturation nadir. Severe OSA is defined as AHI score >10 with an SpO_2_ < 90% for >10% of TST and an oxygen saturation nadir < 80 [14]. Patients were not categorized as mild, moderate, or severe in this study.

### 2.2. Hypertension Classification

Hypertension was defined by the “AAP Clinical Practice Guidelines for Screening and Management of High Blood Pressure in Children and Adolescents.” Hypertension in the pediatric population is the average systolic blood pressure (SBP) and/or diastolic blood pressure (DBP) that is ≥95th percentile for gender, age, and height on ≥3 occasions. For this chart review, the last five BP measurements from clinic visits within the past year prior to polysomnography were averaged. The patient’s height percentile was calculated using the following CDC guidelines for males and females [24]. The calculated height percentile to determine if the patient was hypertensive (≥95th percentile for gender, age, and height) or not hypertensive was used. The blood pressure percentile was determined based on the AAP Clinical Practice Guidelines for Screening and Management of High Blood Pressure in Children and Adolescents. All patients below the 95th percentile in blood pressure for their age, height, and sex were considered non-hypertensive. For this study, those who were diagnosed with hypertension, pre-hypertensive or elevated blood pressure were not included in our hypertensive groups.

### 2.3. Demographic, Social, and Clinical Variables

During our retrospective study, several variables were examined. These included demographic information, family history, and patient history.

Demographic information included: age, gender, race, ethnicity, parents in household, and area deprivation index (ADI, rankings of neighborhoods by socioeconomic disadvantage in a region of interest). Family history included hypertension in the extended family, maternal history of preeclampsia, or gestational hypertension during pregnancy.

Patient history variables included gestational age, birth weight, smoking exposure, number of siblings, orphan status, patient history of surgery, allergic rhinitis, food allergies, BMI z-score (which adjusts for patients age), daytime HR, pubertal status, attention-deficit hyperactivity disorder (ADHD), cardiovascular disease (CVD), diabetes mellitus (DM), renal disease, mental illness, neurological disease, genetic disease, COVID-19, cancer, chronic stimulant use, chronic steroid use, estrogen/testosterone use, and medications for mental illness. Medications for mental illness included selective serotonin reuptake inhibitors (SSRI’s), non-selective serotonin reuptake inhibitors (SNRI’s), antipsychotic medications, and benzodiazepines.

### 2.4. Statistical Analysis

All variables were checked for normality using the Shapiro-Wilk test. Non-parametric (Mann-Whitney Test) two-tailed tests for continuous data and Chi-square tests for categorical data were used to test for significant differences between the non-OSA non-hypertensive and hypertensive individuals. Statistical analysis was performed using MATLAB 2022 (RRID:SCR_001622), GraphPad Prism 10.1 (RRID:SCR_002798), and Microsoft Excel 2020 (RRID:SCR_016137). Data are reported in mean ± standard deviation or percentages rounded to the nearest tenth. *p* < 0.05 is considered statistically significant.

## 3. Results

**Figure 2:**
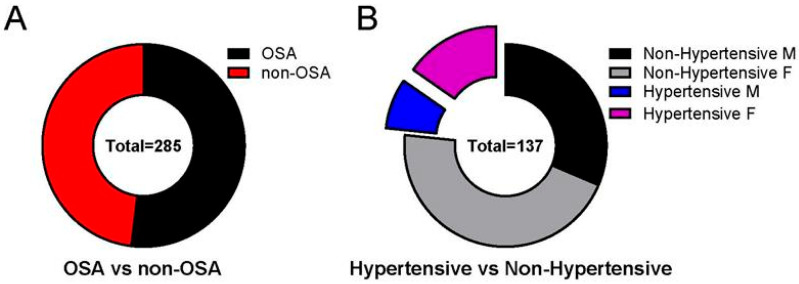
Non-OSA pediatric patients may develop hypertension. Not all patients with hypertension met the criteria of having OSA. (**A**) Of the 285 charts reviewed, 137 did not meet the criteria for OSA based on polysomnography (PSG) (48.07%). (**B**) Of the 137 non-OSA patients, 32 were hypertensive (23.36%). All 32 had systolic hypertension; 2 patients had both systolic and diastolic hypertension. Data is reported as mean ± standard deviation. Two-tailed *t*-test; ** *p* < 0.01, **** *p* < 0.0001.

Of the 285 charts reviewed, 137 did not meet criteria for OSA on PSG (Figure 2) and were classified as non-OSA. Of the 137 non-OSA patients, 83 were females (60.6%) and 54 were males (39.4%). There was no statistically significant difference for sex. Of the 137 non-OSA patients, 32 were hypertensive (23.4%). All 32 had systolic hypertension; 2 patients had both systolic and diastolic hypertension.

Table 1 lists demographics that were found to be statistically significant. There was no difference between the non-hypertensive (NH) and hypertensive (H) group in sex, age, ADI, race, ethnicity, or number of siblings. Family situation (both parents, mother only, extended family, shared custody, no documentation of living situation, Table 1). Table 2 shows that there was no significant difference between the NH group and the H group for surgical history, family history of hypertension, history of preeclampsia, history of gestational hypertension, gestational age at birth, or allergic rhinitis. However, smoking exposure was found to be statistically significant (*p* = 0.009, Table 2) in 10 out of the 105 NH (9.5%) compared to 9 out of 32 H (28.1%). Smoking exposure in the household was defined as one or more other members of the household smoking cigarettes or vaping. Food allergies were found to be statistically significant (*p* = 0.010). Twenty-four out of the one hundred-five NH (22.9%), compared to one out of the thirty-two H (3.1%), were found to have food allergies.

Parents in Household was found to be statistically significant (*p* < 0.0001):69 out of 105 NH (65.714%) compared to 16 out of 32 H (50%) were found to have both parents in the household; 29 out of 105 NH (27.619%) compared to 10 out of 32 (31.25%) were found to have mother only; 1 out of 105 NH (0.952%) compared to 0 out of 32 H (0%) were found to have dad only; 0 out of 105 NH (0%) compared to 2 out of the 32 H (6.25%) were found to be with extended family only; 4 out of the 105 NH (3.810%) compared to the 3 out of 32 H (9.375%) were found to have shared custody; 1 out of the 105 NH (0.952%) compared to 0 out of the 32 H (0%) were found to live in an outdoor facility; 1 out of the 105 NH (0.952%) compared to the 1 out of the 32 H (3.125%) had no documentation of living situation.

Smoking exposure was found to be statistically significant (*p* = 0.009; 10 out of the 105 NH (9.524%) compared to the 9 out of 32 H (28.125%). Food allergies were found to be statistically significant (*p* = 0.010). Twenty-four out of the one hundred-five NH (22.857%) compared to 1 out of the 32 H (3.125%) were found to have food allergies.

**Figure 3:**
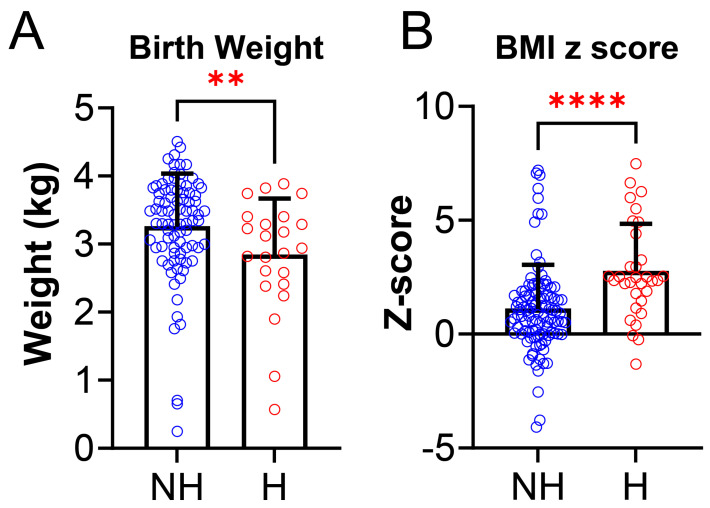
Anthropometric data between non-hypertensives (NH) and hypertensives (H). (**A**) Birth weight was significantly lower in hypertensives [H, (N = 32, red)] compared to non-hypertensives [NH,(N = 105, blue) (NH: 3.25 ± 0.78 kg vs. H: 2.85 ± 0.83 kg, *p* = 0.0097)]. (**B**) BMI z-score was significantly higher in hypertensives (H, N = 32, red) group compared to non-hypertensives (NH, N = 105, blue) group (NH: 1.10 ± 1.94 vs. H: 2.76 ± 2.08, *p* < 0.0001). Data is reported as mean ± standard deviation. Two-tailed *t*-test; ** *p* < 0.001, **** *p* < 0.0001.

**Figure 4:**
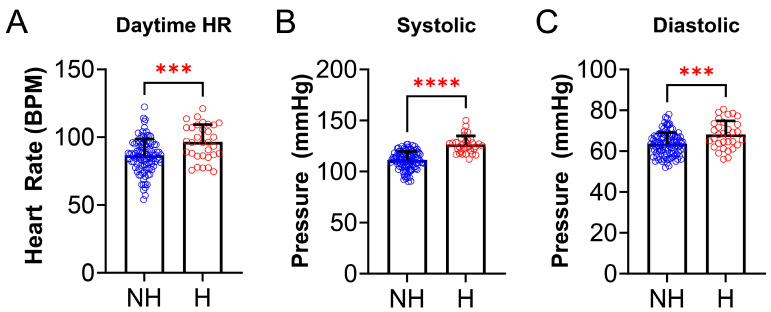
**Cardiovascular parameters differ in non-OSA hypertensives and non-hypertensives.** (**A**) Daytime heart rate (HR), measured at clinic visit, was significantly higher in hypertensives (N = 32, red) compared to the NH group (N = 105, blue) [(NH: 86. 4 ± 12.3 vs. H: 96.4 ± 12.9 beats per minute (BPM), *p* = 0.0004)]. (**B**) Systolic blood pressure (BP), measured at clinic visits, was significantly higher in the H (N = 32, red) group compared to the NH group (N = 105, blue) [(NH: 111.1 ± 8.7 vs. H: 126.2 ± 8.6 mmHg, *p* < 0.0001)]. (**C**) Diastolic BP (measured at clinic visits) was significantly higher in the H (N = 32, red) group compared to the NH group (N = 105, blue) [(NH: 63.4 ± 5.6 vs. H: 68.1 ± 6.7 mmHg, *p* = 0.0006)]. Data is reported as mean ± standard deviation. Two-tailed *t*-test; *** *p* < 0.001, **** *p* < 0.0001.

We also examined anthropometric data and found some statistically significant differences between the NH group and H group. Birth weight was lower in the NH group compared to the H group (NH: 3.25 ± 0.78 kg vs. H: 2.85 ± 0.83 kg, *p* = 0.0097, Figure 3A). There was also a difference between NH and H for BMI (Figure 3B), where BMI z-score was higher in the H group (NH: 1.10 ± 1.94 vs. H: 2.76 ± 2.08, *p* < 0.0001). Daytime HR (NH: 86.39 ± 12.3 vs. H: 126.2 ± 8.6 BPM, *p* = 0.0004, Figure 4A), systolic BP (NH: 63.4 ± 8.7 vs. H: 126.2 ± 8.6 mmHg, *p* < 0.0001, Figure 4B), and diastolic BP (NH: 63.4 ± 5.6 vs. H: 68.1 ± 6.7 mmHg, *p* = 0.0006, Figure 4C), were significantly higher in the H group compared to the NH group (Figure 4).

Certain medical histories were found to be significant as well (Table 3). Among mental illness and developmental disorders, a higher proportion of anxiety was found in 33 out of the 105 NH (31.4%), compared to 6 out of 32 H (18.8%). Similarly, depression was found to be in a higher proportion in the NH group, 15 out of 105 NH (14.3%), compared to 2 out of 32 H (6.3%). Five out of one hundred-five NH (4.8%), compared to one out of thirty-two H (3.1%), were found to have autism. Four out of the one hundred-five NH (3.8%), compared to one out of thirty-two H (3.1%), were found to have mood disorders. Twelve out of the one hundred-five NH (11.4%), compared to two out of thirty-two H (6.3%), were reported to have other mental disorders. Sixty-one out of one hundred-five NH (58.1%), compared to the twenty-three out of thirty-two H (71.9%), reported no mental illness. Among medical disorders, a higher proportion of epilepsy was reported in the NH group, 10 out of 105 NH (9.5%), compared to the H group, 1 out of 32 H (3.1%). Other neurological diseases, which included migraines, cerebral palsy, dysautonomia, structural brain abnormalities, narcolepsy, and developmental delay, were reported at a higher frequency in the NH group, 45 out of 105 NH (42.9%), compared to the H group, 7 out of 32 H (21.9%). However, the presence of no neurological disease was lower in the NH group, 54 out of 105 NH (51.4%), compared to the H group, 25 out of 32 H (78.1%). Asthma was present in a higher proportion in the H group, 16 out of 32 H (50%), compared to the NH group, 36 out of 105 NH (34.3%). Chronic lung disease was present in 7 out of 105 NH (6.7%) vs. 2 out of 32 H (6.3%). Other respiratory disease had 4 out of 105 NH (3.8%) vs. 1 out of 32 H (3.1%); no presence of respiratory illness was reported in 63 out of 105 NH (60%) vs. 14 out of 32 H (43.8%). There were no statistically significant differences between the NH group and H group for history of ADHD, cardiovascular disease, diabetes mellitus, renal disease, or genetic disease.

**Figure 5:**
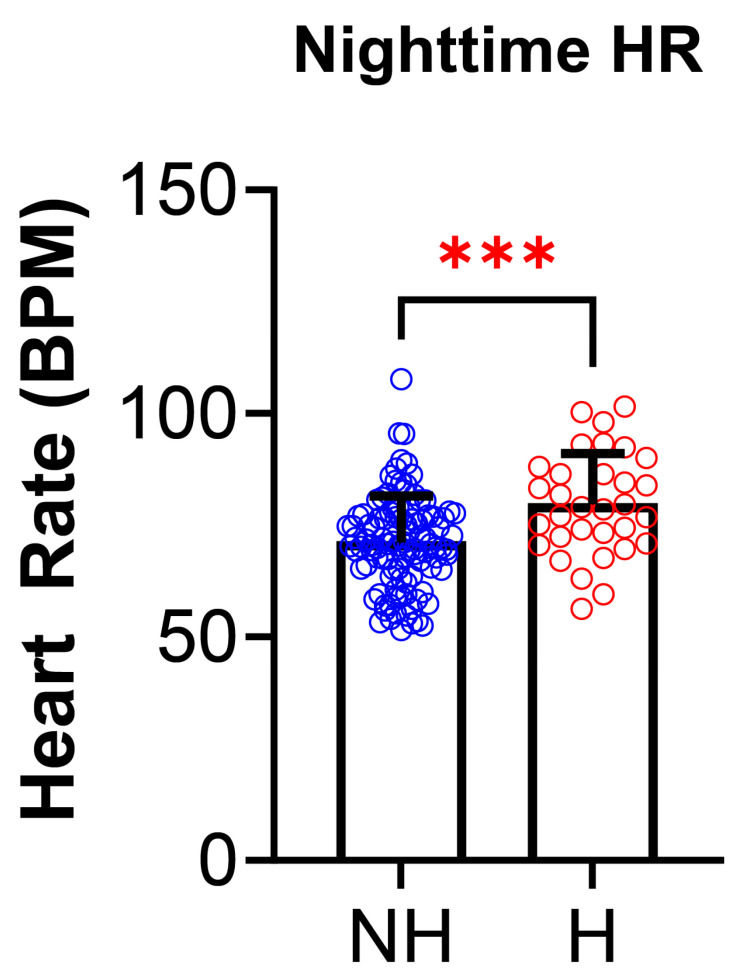
**Nighttime heart rate in polysomnography is increased in those with non-OSA hypertension.** Nighttime heart rate (HR), measured during polysomnography, was significantly higher in hypertensives (H, N = 32, red) compared to non-hypertensives (NH, N = 105, blue) [(NH: 71.2 ± 10.4 vs. H: 79.6 ± 11.5 beats per minute (BPM), *p* = 0.0003)]. Data is reported as mean ± standard deviation. Two-tailed *t*-test; *** *p* < 0.001.

PSG parameters were analyzed for differences between the NH and H groups (Table 4). Of all the PSG parameters, only the nighttime heart rate differed and was higher in the H group than the NH group (NH: 71.2 ± 10.4 vs. H: 79.6 ±11.5 BPM; *p* = 0.0003, Figure 5). There were no statistically significant differences between total sleep time (TST), sleep efficiency (SE), obstructive apnea index (OAI), hypopnea index (HI), apnea index (AI), apnea hypopneas index (AHI), O_2_ saturation nadir, %O_2_ below 90%, nocturnal hypoxemia, baseline EtCO_2_, %TST EtCO_2_ >50, hypoventilation, TST snoring, percent time in N1, N2, N3, or R, REM AHI, arousal index, WASO, or PLM index between the two groups.

Nighttime heart rate (HR) was the only variable found to be statistically significant with the H group having a significantly higher nighttime HR (*p* = 0.0003, 71.2 ± 10.4 NH vs. 79.6 ± 11.5 H). There were no statistically significant differences in the other PSG parameters. The differences in medication usage between the NH group and the H group (Table 5) were also assessed. There were no statistically significant differences between the NH group and H group for use of systemic steroid use, stimulant use, or medications for mental illness.

A higher proportion of anxiety was found in 33 out of the 105 NH (31.429%) compared to 6 out of 32 H (18.75%). Similarly, depression was found to be in a higher proportion in the NH group, 15 out of the 105 NH (14.286%) compared to the 2 out of 32 H (6.25%). Five out of the one hundred-five NH (4.762%) compared to one out of thirty-two H (3.125%) were found to have autism. Four out of one hundred-five NH (3.810%) compared to one out of thirty-two H (3.125%) were found to have mood disorders. Twelve out of one hundred-five NH (11.423%) compared to the two out of thirty-two H (6.25%) were reported to have other mental disorders. Sixty-one out of one hundred-five NH (58.095%) compared to twenty-three out of thirty-two H (71.875%) reported no mental illness. A higher proportion of epilepsy was reported in the NH group, 10 out of 105 NH (9.524%), compared to the H group, 1 out of 32 H (3.125%). Other neurological diseases were reported at a higher frequency in the NH group, with 45 out of 105 NH (42.857%), compared to 7 out of 32 in the H group (21.875%). No neurological disease was reported lower in the NH group, 54 out of 105 NH (51.429%), compared to the H group, 25 out of 32 H (78.125%). Asthma had a higher proportion in the H group, 16 out of 32 H (50%), compared to the NH group, 36 out of 105 NH (34.286%). Chronic lung disease had 7 out of 105 NH (6.667%) vs. 2 out of 32 H (6.25%). Other respiratory disease had 4 out of 105 NH (3.810%) vs. 1 out of 32 H (3.125%). No respiratory illness was reported 63 out of 105 NH (60%) vs. 14 out of 32 H (43.75%).]

There were no statistically significant differences between the NH group and H group for systemic steroid use, stimulant use, and medications for mental illness.

## 4. Discussion

In our study, we evaluated factors associated with pediatric hypertension in the absence of OSA. Interestingly, of the 285 charts reviewed, 137 did not meet the criteria for OSA on PSG in this referenced population. Of the 137 non-OSA patients, 60.6% were female and 39.4% male. Of the 137 non-OSA patients, 23.7% were hypertensive. All had systolic hypertension; 2 patients had both systolic and diastolic hypertension.

### 4.1. Sociodemographic Characteristics

Among demographics, we expected but did not observe a difference between the NH and H group in terms of ADI and race. Previous studies have shown that residing within communities with ADI greater than or equal to 50 was associated with 60% greater odds of a hypertension diagnosis [25]. However, the study was based on the state percentile while others have utilized national percentiles, which could explain why there is not a notable difference in ADI. The small sample size may also be a reason for no statistically significant difference, indicating that larger studies are necessary to assess the relationship. Previous studies have also shown differences in race for pediatric hypertension. The crude prevalence of hypertension was significantly higher in non-Hispanic Blacks compared to non-Hispanic White youth, but not in Mexican Americans. In stratified analysis by age-sex groups, the Black-White difference in hypertension prevalence was only significant among boys aged 13–17. After controlling for age, Black boys had 51% higher odds of having hypertension compared to White youth at ages 13–17 [26]. This racial difference persisted with additional adjustment for birth weight and for current body mass index. In the study, there was not an observable significant difference due to sample size and/or lack of stratification. However, there was a statistically significant difference in birth weight between NH and H groups. Prior studies have found that birth weight was negatively correlated with SBP, even after adjusting for the relevant covariates [27]. Although the study showed that the H group had an average birth weight greater than 2500 g, which is the threshold for low birth weight determined by the World Health Organization, a similar relationship is still seen, and this medical history may warrant additional follow up to mitigate the risk of developing hypertension.

### 4.2. Heart Rate and Blood Pressure

The study found that daytime and nighttime HR was significantly higher in the hypertensive group (Figure 4 and Figure 5). Studies to date suggest a role of cardiac over activity, characterized by increased heart rate and left ventricular ejection, and increased aortic stiffness as the main hemodynamic determinants of primary hypertension in children [28]. It is also possible that nighttime HR could be elevated due to the PSG being performed in a sleep lab which can induce first night effect or anxiety due to being in unfamiliar surroundings. For patients with a history of anxiety, it may be beneficial to perform a home portable PSG instead of at a facility, but home studies are not yet FDA approved as standard testing for sleep related breathing disorder in pediatric patients. There is a statistically significant difference in birth weight between NH and H; with the hypertensives having a lower birth weight. The findings of smoking exposure were statistically significant, indicating association between smoke exposure and hypertension, as seen in prior studies [29,30,31]. Data from the Bogalusa Heart study showed that resting HR was positively correlated with both markers of obesity and BP [32]. It was found that HR was highest in obese hypertensive schoolchildren, and lowest in normotensive, non-obese children [3,33]. Elevated HR during sleep may suggest that obesity and sympathetic activation are important in the pathogenesis of pediatric hypertension, even in the absence of detectable sleep related breathing disorders. Future studies with larger cohorts are necessary to further explore these relationships.

### 4.3. Parents in Household

Currently, no studies are available that assess the relationship between hypertension and the presence of parents in the household. Prior studies have found that anxiety has been linked to pediatric hypertension [34]. Although the cause of the anxiety is unclear, deviations from the nuclear family structure have negative effects on the mental well-being of children [35]. In our study, parents in the household were found to be a significant factor; specifically, the hypertensive group had a lower proportion of both parents in the household compared to the non-hypertensive group.

### 4.4. Food Allergies

Food allergies were found to be statistically significant, with non-hypertensives having a higher percentage of food allergies. There are currently no published studies demonstrating the relationship between food allergies and pediatric hypertension. However, previous studies suggest that food allergies, especially in the setting of anaphylaxis, are associated with hypotension in all ages [36].

### 4.5. Medical History

Medical histories of mental illness, neurological diseases, and respiratory diseases were found to differ between the non-hypertensives and hypertensives. Interestingly, those in the NH group had a higher proportion of anxiety compared to the H group, contradicting previous findings that show a relationship between elevated blood pressures and anxiety in adolescent patients [34]. There may have been this relationship due to grouping pre-hypertensive patients with normotensive patients. Future studies should aim to have a normotensive, pre-hypertensive, and hypertensive group. Additionally, patients who may not have a diagnosis of chronic anxiety may have had acute anxiety due to the office visit and the elevated blood pressure may be a result of whitecoat hypertension [37]. Similarly, depression and mood disorders were found in a higher proportion in the NH group compared to the H group. This may also be a result of combining pre-hypertensive patients with normotensive patients. Neurological diseases were also found to be statistically significant. A higher proportion of epilepsy was reported in the NH group compared to the H group. According to a study by Wilner et al., in patients 19 years and older with epilepsy, hypertension was the most common comorbid condition [38]. The discrepancy between our study and Wilner et al. highlights the importance of further studies to fully elucidate the relationship between hypertension and epilepsy. In addition, our study found that other neurological diseases were reported at a higher frequency in the NH group compared to the H group (Table 3). Asthma was found to be significantly higher in hypertensives than non-hypertensives. Prior studies showed that the age- and covariate-adjusted prevalence of obesity, dyslipidemia, arthritis, diabetes, and hypertension is higher in adult-onset asthma than in childhood-onset asthma, and with older age of asthma diagnosis. Conversely, the prevalence of chronic obstructive pulmonary disease increases with younger age of asthma diagnosis [39]. There were no statistically significant results in this study regarding medication use. Although prior studies have shown that stimulants, some antidepressants, systemic corticosteroids, estrogens, androgens, and oral contraceptives may increase blood pressure [40].

### 4.6. PSG Parameters

Previous studies have shown that one PSG parameter, REM AHI, has significant dose-relationships between REM AHI and prevalent hypertension. In individuals with non-REM AHI less than or equal to 5, a twofold increase in REM AHI was associated with 24% higher odds of hypertension (odds ratio, 1.24; 95% confidence interval, 1.08–1.41). Longitudinal analysis revealed a significant association between REM AHI categories and the development of hypertension [41]. Upper airway muscle activation is usually thought to be more severely suppressed during REM sleep than during NREM sleep. It is believed that due to the suppression of upper air muscle, activation during REM contributes to the repeated episodes of sleep apnea and nocturnal episodic hypoxemia [42,43,44]. Several studies have shown that longer duration of apnea, more severe hypoxemia, and apnea-induced desaturations dropped more after an obstructive apnea in REM sleep than NREM sleep [45,46]. Although the study did not show differences between NH and H, the H trended higher REM AHI compared to the NH group REM AHI, which may be due to the H group having a larger BMI and more likely to have obstructed airways. Although there is a trend, the results may not have been significant due to our small sample size.

Interestingly, in our study, the PLM index did not differ between the NH and H groups. An adult systematic review and meta-analysis of observational studies reported the pooled risk ratio of 1.26 for hypertension in patients with PLMS (95% CI, 1.12–1.41) [47]. In a cross-sectional study, children with PLMS were at significantly higher risk for nocturnal systolic (adjusted OR (95%CI) = 6.25 [1.87–20.88]) and diastolic (OR (95%CI) = 4.83 [1.66–14.07]) hypertension. In the same study, there was a trend toward higher daytime BP in children with PLMS compared to those children without PLMS (*p* = 0.084 for systolic BP z score; *p* = 0.051 for diastolic BP z score; *p* = 0.067 for systolic pre-hypertension) [48]. Although our study did not show statistical significance, the H group had a higher PLM index compared to the NH group. The lack in statistical significance could be due to our sample size, so larger studies are warranted to further evaluate the relationship between PLM index and hypertension.

This study begins to identify invaluable information on the development of hypertension in children without OSA. Despite the important information derived from our study, there are some limitations of the study. First, the observational design inherently restricts the ability to draw causal inferences. Blood pressure measurements were also taken at different times of the day which may have led to blood pressure variability across patients. Second, the sample size was relatively small, which may limit the generalizability of the findings. Additionally, multiple reviewers were involved in chart abstraction, potentially introducing inter-rater variability; however, this was addressed through regular meetings and the implementation of standardized chart review criteria. Finally, the use of the Area Deprivation Index (ADI) was limited to state-level data, rather than utilizing national-level data, which may have impacted the comprehensiveness of socioeconomic assessments. However, this study provides a direction in which future studies can explore the factors involved with hypertension in children without OSA.

## 5. Conclusions

Our study thus suggests that pediatric patients, even in the absence of OSA, can develop hypertension. Parents in household, presence of food allergies, smoking exposure, low birth weight, elevated BMI, elevated daytime and nighttime heart rate, and certain medical conditions such as mental illness, respiratory diseases, and neurological diseases were associated with hypertension in non-OSA pediatric patients. These findings provide insight on the development of hypertension in the pediatric population outside of OSA and social factors may warrant additional follow up in the screening of hypertension in pediatric patients. More studies with larger sample sizes and longitudinal studies are needed to further investigate the risk factors associated with pediatric hypertension to help mitigate serious health concerns.

## Figures and Tables

**Table 1:** Demographics of pediatric patients with non-OSA presenting non-hypertensive (NH) or hypertensive (H).

	Non-Hypertensive (n = 105)	Hypertensive (n = 32)	*p*-Value
**Sex**	Male (n = 43), Female (n = 62)	Male (n = 11), Female (n = 21)	0.505
**Age**	12.6 ± 2.9 years	11.5 ± 2.8 years	0.052
**ADI**			0.706
1	18	7	
2	16	3	
3	12	5	
4	8	3	
5	9	1	
6	5	0	
7	5	3	
8	6	2	
9	13	6	
10	13	2	
**Race**			0.322
White	66	16	
Black	28	11	
Hispanic	1	0	
Asian	5	0	
Other	5	5	
**Ethnicity**			0.861
Black	1	0	
Hispanic	13	4	
Non-Hispanic	90	27	
Other	0	1	
Not Documented	1	0	
**Parents in Household ^#^**			***<0.0001***
Both Parents	69 (4 with extended family as well)	16	
Mom Only	29 (4 with extended family as well)	10 (4 with extended family as well)	
Dad Only	1	0	
Extended Family Only	0	2	
Shared	4	3	
Outside Facility	1	0	
Not Documented	1	1	
**# of Siblings**			0.185
0	17	10	
1	37	9	
2	30	7	
3	13	2	
4	3	2	
5	3	1	
6	1	0	
Not documented	1	1	

**^#^** Including those who lived with extended family as well.

**Table 2:** Personal and family history of certain exposures in pediatric patients with non-OSA presenting non-hypertensive (NH) or hypertensive (H).

	Non-Hypertensive (n = 105)	Hypertensive (n = 32)	*p*-Value
**Surgical History ^##^**			0.408
Adenoidectomy	5 (3 with other surgeries)	5 (4 with other surgeries)	
Tonsillectomy	1	0	
Adenoidectomy + Tonsillectomy	21 (5 with other surgeries)	6 (2 with other surgeries)	
Other Surgery Only	23	2	
None	55	18	
Not Documented	0	1	
**Family Hx of HTN ^###^**			0.691
Yes	37	14	
Who?			
Father	12	4	
Mother	3	3	
Both Parents	1	1	
Sibling	0	1	
Extended Family	25	11	
Unsure	1	0	
No	68	17	
Not Documented	0	1	
**Hx of Preeclampsia**			0.164
Yes	2	2	
No	95	25	
Not Documented	8	5	
**Hx of Gestational HTN**			0.727
Yes	2	0	
No	95	26	
Not Documented	8	6	
**Gestational Age at Birth**			0.880
Full Term	83	26	
Premature	14	4	
Not Documented	8	2	
**Smoking Exposure**			***0.009***
Yes	10	9	
No	93	23	
Not Documented	2	0	
**Allergic Rhinitis**			0.404
Yes	37	14	
No	67	18	
Not Documented	1	0	
**Food Allergies**			***0.010***
Yes	24	1	
No	80	31	
Not Documented	1	0	

**^##^** Including those who had multiple surgeries. **^###^** Including those who had multiple family members with a history of hypertension.

**Table 3:** Medical history of pediatric non-OSA with and without hypertension.

	Non-Hypertensive (n = 105)	Hypertensive (n = 32)	*p*-Value
**ADHD**			0.985
Yes	33	10	
No	72	22	
**Cardiovascular Disease**			0.917
Congenital Heart Disease	10	3	
Heart Failure	0	0	
Cardiac Surgery	0	0	
Other CVD	5	3	
None	90	26	
**Diabetes Mellitus**			0.194
Type 1	0	0	
Type 2	0	1	
None	105	31	
**Renal Disease**			
CKD Stage 1	2	1	0.997
CKD Stage 2	0	0	
CKD Stage 3	0	0	
CKD Stage 4	0	0	
CKD Stage 5	0	0	
Dialysis Dependent	0	0	
None	103	31	
**Mental Illness ^^^**			***<0.0001***
Anxiety	33	6	
Depression	15	2	
Autism	5	1	
Mood Disorder	4	1	
Other	12	2	
None	61	23	
**Neurological Disease ^^^^**			***0.03***
Epilepsy	10	1	
Other	45	7	
None	54	25	
**Genetic Disease**			0.25
Achondroplasia	1	0	
Prader-Willi	0	2	
Down Syndrome	0	0	
Sickle Cell Disease	1	2	
Other	17	3	
None	86	26	
**Respiratory Disease ^^^^^**			***<0.0001***
Asthma	36	16	
Chronic Lung Disease	7	2	
Cystic Fibrosis	0	0	
Other	4	1	
None	63	14	

**^^^** 25 NH and 5H had multiple mental illness listed; **^^^^** 4 NH and 1H had multiple neurological diseases listed; **^^^^^** 5 NH and 1H had multiple respiratory diseases listed.

**Table 4:** Sleep Parameters of Pediatric Non-OSA With and Without Hypertension.

	Non-Hypertensive (n = 105)	Hypertensive (n = 32)	*p*-Value
**Total Sleep Time**	385.8 ± 58.9	363.9 ± 84.3	0.1797
**Sleep Efficiency (SE)**	78.4 ± 12.3	76.0 ± 11.7	0.2628
**Obstructive Apnea Index (OAI)**	0.86 ± 8.1	0.063 ± 0.11	0.4384
**Hypopnea Index (HI)**	2.1 ± 1.9	1.8 ± 1.5	0.4819
**Apnea Index (AI)**	0.62 ± 0.74	1.0 ± 2.0	0.9684
**Apnea Hypopneas Index (AHI)**	2.7 ± 2.2	2.8 ± 2.9	0.5205
**O_2_ Saturation Nadir**			
**%O_2_ below 90%**	0.006 ± 0.05	0.05 ± 0.29	0.1853
**Nocturnal Hypoxemia**			1
**Baseline EtCO_2_ (mmHg)**	41.2 ± 7.0	42.6 ± 2.8	0.4146
**%TST EtCo_2_ > 50**	7.2 ± 17.8	9.8 ± 19.0	0.2862
**Hypoventilation**			0.427
**TST Snoring (%)**	25.8 ± 19.8	28.2 ± 19.4	0.5733
**Percent Time in**			
N1	3.8 ± 3.5	3.5 ± 2.5	0.7894
N2	53.9 ± 8.8	51.5 ± 9.2	0.1376
N3	25.9 ± 10.1	28.5 ± 9.6	0.2152
R%	16.5 ± 6.7	16.3 ± 6.1	0.8663
**REM AHI**	3.6 ± 3.6	5.3 ± 5.5	0.2564
**Arousal Index**	12.5 ± 6.3	14.6 ± 10.4	
**WASO**	46.8 ± 38.9	57.8 ± 52.1	0.4996
**OSAS**			1.000
**Nighttime HR**	71.2 ± 10.4	79.6 ± 11.5	***0.0003***
**PLM Index**	2.3 ± 5.4	3.0 ± 6.9	0.1922

**Table 5:** Medication usage of pediatric non-OSA with and without hypertension.

	Non-Hypertensive (n = 105)	Hypertensive (n = 32)	*p*-Value
**Systemic Steroid Use**			0.91
Yes	22	7	
No	83	25	
**Stimulant Use**			0.878
Yes	31	9	
No	74	23	
**Medication for Mental Illness**			0.194
Yes	32	6	
No	73	26	

## Data Availability

All data is available upon reasonable request to the corresponding author (DM).
